# Pathogenesis of Uveitis in Ebola Virus Disease Survivors: Evolving Understanding from Outbreaks to Animal Models

**DOI:** 10.3390/microorganisms8040594

**Published:** 2020-04-20

**Authors:** Caleb Hartley, J. Clay Bavinger, Sanjana Kuthyar, Jessica G. Shantha, Steven Yeh

**Affiliations:** 1 Department of Global Health, Rollins School of Public Health, Emory University, Atlanta, GA 30322, USA; caleb.hartley@emory.edu; 2 Department of Ophthalmology, University of Pennsylvania, Philadelphia, PA 19104, USA; claybavinger@gmail.com; 3 Department of Ophthalmology, Emory University, Atlanta, GA 30322, USA; sanjana.kuthyar@emory.edu; 4 Emory Global Health Institute, Emory University, Atlanta, GA 30322, USA

**Keywords:** Ebola virus disease, uveitis, animal models, outbreaks, emerging infectious diseases

## Abstract

Ebola virus disease (EVD) and emerging infectious disease threats continue to threaten life, prosperity and global health security. To properly counteract EVD, an improved understanding of the long-term impact of recent EVD outbreaks in West Africa and the Democratic Republic of Congo are needed. In the wake of recent outbreaks, numerous health sequelae were identified in EVD survivors. These findings include joint pains, headaches, myalgias, and uveitis, a vision-threatening inflammatory condition of the eye. Retrospective and more recent prospective studies of EVD survivors from West Africa have demonstrated that uveitis may occur in 13–34% of patients with an increase in prevalence from baseline to 12-month follow-up. The clinical spectrum of disease ranges from mild, anterior uveitis to severe, sight-threatening panuveitis. Untreated inflammation may ultimately lead to secondary complications of cataract and posterior synechiae, with resultant vision impairment. The identification of Ebola virus persistence in immune privileged organs, such as the eye, with subsequent tissue inflammation and edema may lead to vision loss. Non-human primate models of EVD have demonstrated tissue localization to the eye including macrophage reservoirs within the vitreous matter. Moreover, in vitro models of Ebola virus have shown permissiveness in retinal pigment epithelial cells, potentially contributing to viral persistence. Broad perspectives from epidemiologic studies of the outbreak, animal modeling, and immunologic studies of EVD survivors have demonstrated the spectrum of the eye disease, tissue specificity of Ebola virus infection, and antigen-specific immunologic response. Further studies in these areas will elucidate the mechanisms of this highly prevalent disease with the potential for improved therapeutics for Ebola virus in immune-privileged sites.

## 1. Introduction

Ebola virus disease (EVD) was first discovered in the Democratic Republic of Congo (DRC) in 1976 [1]. Since then, sporadic outbreaks have occurred primarily in rural DRC while other outbreaks have occurred in Uganda, Gabon, Sudan, and Republic of Congo. Prior to the West African EVD outbreak, which started in late 2013, only 2345 confirmed cases had been observed [2]. The magnitude of the West African EVD outbreak from 2013 to 2016, primarily within the highest transmission countries of Sierra Leone, Liberia and Guinea, eclipsed all prior outbreaks combined by over 10-fold with over 28,600 cases and 11,300 deaths [3]. While this outbreak was unprecedented in scope and magnitude, ultimately with 12 countries affected and threatening health security globally, lessons learned from the West African EVD outbreak were translated to more recent EVD outbreaks in DRC.

Since the end of the West African EVD outbreak, there have been three additional outbreaks in the DRC, one of which remains ongoing in conflict-ridden, eastern DRC. This ongoing outbreak is the second largest EVD outbreak in history, with World Health Organization reports of 3444 current cases in the DRC epidemic with 2264 deaths and 1167 EVD survivors [4]. 

In the wake of the West African EVD outbreak and recent DRC outbreak, the international community has recognized that thousands of survivors are at risk of post EVD sequelae. These system health sequelae include uveitis, arthritis/arthralgias, psychosocial stressors and mental health disorders, as well as viral persistence in immune privileged organs (e.g., eye, reproductive organs, and central nervous system). 

Uveitis, in particular, has been reported with a high prevalence, ranging from 13–34% [5,6,7]. A recent prospective study in Liberia has demonstrated that uveitis may be observed in 26% of EVD survivors compared to 12% of control patients [8]. The spectrum of disease ranges from anterior uveitis to panuveitis, with sight-threatening complications in nearly 40% of individuals who develop uveitis [6]. Ebola viral persistence has been identified in association with a severe sight-threatening panuveitis in an EVD survivor, illustrating the potential for organ-threatening disease despite systemic clearance [9]. 

In this review, we discuss the ophthalmic spectrum of the disease, mechanisms of uveitis and eye disease in EVD survivors, which are illustrative of the balance between lytic viral infection and inflammatory mechanisms. Epidemiologic observations and animal models have contributed to our understanding of Ebola virus persistence in the eye. However, further investigation is needed to fully understand the balance between infection and immunity in this highly prevalent disease condition. 

## 2. General Considerations and Ophthalmic Findings in Acute Ebola Virus Disease (EVD) 

*Ebolavirus* is an enveloped, non-segmented, single-stranded RNA virus [10]. Five species of Ebola virus have been identified, and the genus *Ebolavirus* belongs to the *Filoviridae* family [2]. *Zaire ebolavirus* is the most severe strain and has been associated with the highest case fatality rate [2]. Potential animal reservoir hosts include fruit bats, non-human primates (chimpanzees, gorillas), duikers and various rodents [11]. Animal-to-human transmission led to the suspected index case of EVD in Guinea in late 2013 [12]. 

The clinical features of acute EVD include fever, fatigue, abdominal pain, vomiting, diarrhea, myalgia and hemorrhage. Hypovolemic shock due to increased vascular permeability and severe diarrhea is considered the most fatal aspect of the disease [2]. Ophthalmic findings that have been reported during acute EVD include vision loss of undetermined etiology, subconjunctival hemorrhage and conjunctivitis [13]. During the West African EVD outbreak, a United States health care worker repatriated to the National Institutes of Health was found to have severe panuveitis shortly after clearance of Ebola virus (EBOV) RNA by reverse transcriptase polymerase chain reaction (RT-PCR) testing [14]. 

Following acute EVD, survivors may experience a number of sequelae, which has been termed the post-Ebola virus disease syndrome (PEVDS), which can include arthralgia, myalgia, hearing loss and tinnitus, cognitive impairment, and ocular disease [7].

## 3. Ophthalmic Manifestations: Key Sequelae of EVD

Prospective and retrospective studies have shown that ocular disease is commonly observed in EVD survivors (Table 1). Disease findings may include uveitis, cataract, and optic neuropathy, with uveitis consistently described as the most common manifestation, occurring in up to one-third of individuals in two reports [5,8]. Symptoms of uveitis, or ocular inflammation, include photophobia, blurred vision, and visual floaters, which were described by EVD survivors [6].

**Figure 1:**
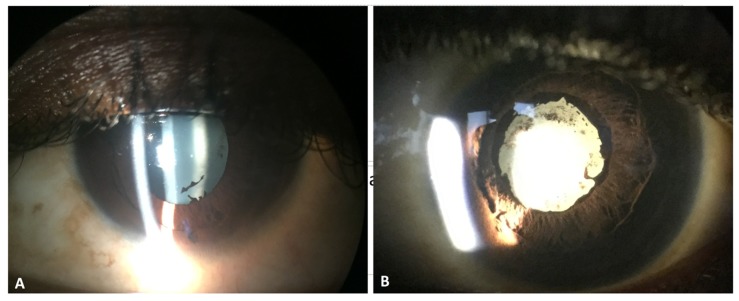
Slit lamp photographs of Ebola virus disease (EVD) survivors shows mild anterior uveitis with posterior synechiae at 5 o’clock and pigment on the lens capsule (**A**). Slit lamp photograph shows an EVD survivor with severe panuveitis who developed diffuse posterior synechiae, pigment on the anterior lens capsule and dense white cataract, likely owing to multiple recurrences of uveitis (**B**).

We previously evaluated nearly 100 EVD survivors who were studied in Liberia, and we identified uveitis in 21 of 96 patients (22%) [6]. Among eyes with EVD-associated uveitis, 38% met the World Health Organization definition of blindness, with a visual acuity worse than or equal to 20/400 [6]. Causes of vision loss included cataract and chorioretinal scarring (Figure 1), both sequelae of uveitis, as well as limited cases of optic neuropathy. 

A more recent, natural history study of 1000 EVD survivors in Liberia found a similar prevalence of uveitis (26% on initial exam, 33% at one-year follow-up), but reported moderate to severe vision loss in only 2.5–3.1% of subjects [8]; 18% of patients retrospectively evaluated in an EVD survivor clinic in Port Loko, Sierra Leone showed signs of uveitis, although visual acuity impairment was not specifically documented [15]. Furthermore, a report of 340 EVD survivors in Guinea described uveitis in 13.5% of patients, with 15 subjects experiencing vision loss to 20/40 or worse in at least one eye [7]. Taken together, the evidence suggests that uveitis is a common ocular manifestation of EVD with the potential for severe vision loss. While the range of vision loss may vary, further studies related to the variations of uveitis and risk factors that can lead to vision impairment are needed. These data are particularly relevant to resource-limited settings, given the impact to economic productivity and quality-of-life reduction associated with vision impairment. 

Signs of uveitis, which have been observed in EVD survivors, include keratic precipitates, posterior synechiae, uveitic cataract, iris heterochromia, and chorioretinal scarring [6]. Besides findings of uveitis, neuro-ophthalmologic findings have included optic atrophy with resultant color vision deficits, ocular motility disturbances and nystagmus [6]. 

## 4. Ophthalmic Findings and Viral Persistence in Animal Models of EVD

Non-human primate models of sequelae following acute EVD have provided insight into the disease manifestations, tissue localization of Ebola virus, and dynamics of Ebola virus entry into immune privileged organs. A delayed time-to-death macaque model with experimental Zaire Ebola virus infection showed viral antigen in the brain, eye, pancreas, and lung [18]. In another Rhesus macaque non-human primate (NHP) with apparent recovery following Ebola virus infection, necrotizing scleritis, conjunctivitis, and optic neuritis were observed with histologic lesions that demonstrated strong Ebola virus antigen staining [19]. The pathologic changes described in this NHP showed patterns reminiscent of eye disease observed in West African EVD survivors. 

More recently, Zeng et al. evaluated persistent, asymptomatic Ebola virus infection in rhesus monkeys that survived EVD infection after treatment with medical countermeasures [20]. Progressive EBOV dissemination into the eyes, brains, and testes were observed. Notably, CD68+ macrophages in the vitreous humor were identified as an EBOV reservoir, with localization largely at the vitreoretinal interface [20]. Whether EBOV may reside in the vitreous cavity of patients is unknown, as EBOV has only been identified in the aqueous humor of an EVD survivor [9]; however, further investigation in this area is needed. An in vitro study of retinal pigment epithelial (RPE) cells showed that RPE was permissive to Ebola virus infection and supported viral replication, and subsequent release of virus in high titers [21]. These findings suggest that the RPE is a potential ocular reservoir, with the potential for persistence of live EBOV within the eye. RPE hyperpigmentation and multifocal chorioretinal scarring have been observed both in United States and West African EVD survivors [6,22].

Besides the eye, other immune privileged sites where Ebola virus persistence has been documented, with resultant clinical implications, include the central nervous system and reproductive organs [17,21,23]. Meningoencephalitis has been reported as a late sequela in an Ebola survivor [14] and sexual transmission was reported in association with viral persistence in West Africa [23,24]. 

## 5. Diagnosis and Immunologic Response

The most common laboratory tests used to diagnose EVD are RT-PCR and enzyme-linked immunosorbent assay (ELISA) testing for Ebola virus antigen or host antibody response [25]. RT-PCR has become the preferred technique due to improved sensitivity and increased availability during acute EVD outbreaks [26].

Given the potential for EBOV to persist or show delayed clearance in specific tissues, RT-PCR has been utilized in the assessment for EBOV in seminal fluid, breast milk, and ocular fluid samples [27]. Detection of EBOV in breast milk and sexual transmission were reported during the West African EVD outbreak [24,28]. In addition, delayed clearance of EBOV from the conjunctiva has also been documented [27]. While the precise clinical significance of delayed clearance of EBOV from specific tissues is unclear, further studies into the clearance dynamics and potential for viral transmission are needed. 

Genetic testing of the 2013–2016 West African outbreak confirmed that it was due to the Zaire strain of EBOV, the same strain responsible for many of the previous known human Ebola outbreaks, which resulted in far fewer cases [29]. However, one potential reason for increased infectivity in recent outbreaks was explored in a study evaluating a glycoprotein (GP) mutation that interfaces with the receptor for EBOV in human cells. This mutation has been shown to increase infectivity in humans and other primates, but decreases infectivity in fruit bats, suggesting that the mutation was an adaption to human hosts [30,31]. This mutation, known as A82V, appeared in early 2014 and was found in more than 90% of subsequent genomic sequencing [30]. Whether there have been mutations associated with greater mortality is controversial, with some results suggestive of an increased mortality in recent strains of EBOV [30]. Other studies report no increase in mortality or even decreased mortality [29,32]. 

EBOV employs several tactics to avoid and alter the standard human immune response to viral infection. While cell entry would otherwise be difficult for a large viral particle, EBOV uses apoptotic mimicry to induce cellular uptake in human phagocytic cells by appearing as an apoptotic body [33,34]. EBOV also disrupts both interferon signaling [35], and RNA inhibition [36,37], both of which are important components of the human antiviral defense mechanism. In addition, a significant decrease in cell-mediated immunity may occur via death of antigen-presenting cells [33] and lymphocyte apoptosis [38]. An antibody decoy may also disrupt humoral immunity [39]. Studies of the immune responses of patients who survive and those that die of acute EBV have consistently shown that survivors exhibit higher numbers of T-cells, specifically CD8+ T-cells, likely making them better able to counteract viral disruption of cell-mediated immunity [40]. Within the eye, RPE cells infected by EBOV in vitro express immunomodulatory molecules linked to ocular immune privilege [21]. 

While these studies illustrate the mechanisms of viral escape systemically and within the eye, the precise trigger for EBOV replication and lytic infection is unknown and raises questions related to its mechanism. Strong et al. described their model system in which EBOV infection of bat and mouse cell lines led to viral persistence and low-level infection [41]. Lipopolysaccharide (LPS) or phorbol-12-myristate-13-acetate (PMA) can lead to reactivation of EBOV and increased replication via the Ras/MAPK-dependent pathway. This presumptive mechanism may be responsible for EBOV emergence from animal reservoirs and transmission to human and/or nonhuman primates during the initiation of EVD outbreaks [41]. 

While it is unknown whether similar pathways could be activated in the transition from EBOV persistence in the eye to more productive infection, the variable timing of uveitis onset in patients, and acute nature of the presentation of EVD survivors who develop uveitis suggest that these mechanisms warrant further exploration. 

## 6. Medical and Surgical Management of Ophthalmic Sequelae of EVD

Our understanding of uveitis related to EVD has continued to evolve, with corresponding modifications to treatment based on the balance between EBOV infection and inflammatory mechanisms. In addition, the anatomic location of uveitis (i.e., anterior uveitis involving the iris and ciliary body; posterior uveitis involving the retina, RPE and choroid) may require different strategies owing to varying pharmacokinetics related to drug penetration into target tissues. 

During our acute treatment of an EVD survivor with Ebola virus persistence, the oral antiviral favipiravir was utilized to treat the infection [22]. However, a combination of topical corticosteroid for anterior uveitis and systemic corticosteroid were utilized to treat the intermediate uveitis (i.e., ciliary body and vitreous inflammation) and scleritis that ensued as the disease progressed. Moreover, a local periocular corticosteroid injection was eventually administered given the progressive inflammation and hypotony (i.e., extremely low intraocular pressure) that developed despite early measures with antiviral, oral and topical corticosteroids [22]. 

The medical management of EVD-associated uveitis has been premised on the anatomic location of disease. Cases of anterior uveitis have been treated with topical corticosteroids [10]. More posterior locations of disease (i.e., intermediate, posterior, panuveitis) have required systemic corticosteroid in conjunction with antiviral therapy [9,42].

Invasive surgery, used to best address cataracts, requires circumspect consideration for protection of the healthcare provider team including ophthalmologist, the patient, and their providers due to potential EBOV persistence in ocular fluid. A cataract has been observed in 5–10% of EVD survivors with uveitis [6], but other ophthalmic diseases unrelated to uveitis, which may also be observed in EVD survivors that require surgery (e.g., retinal detachment, age-related cataract), also have raised questions regarding safety in tissues and fluids that may harbor virus. 

To address this question, we designed the Ebola Virus Persistence in Ocular Tissues and Fluids (EVICT) Study [17]. In this study, 50 EVD survivors were recruited for ocular fluid sampling, the majority of whom required cataract surgery. Forty-nine aqueous humor samples and 1 vitreous sample tested negative for EBOV by RT-PCR, allowing patients to proceed with manual small incision cataract surgery (MSICS), the preferred surgery for many resource-limited settings. The median time of ocular fluid assessment was 19 months during the first phase of testing, and 34 months during the second phase of testing. The cataract surgical results were also promising with the majority of patients achieving 20/40 or better vision at follow-up [17]. This study provided evidence of the safety, feasibility, and vision restorative capability of cataract surgery in EVD survivors, which are a key component of patient health and recovery. 

However, beyond these considerations, questions remain related to the timing of clearance of virus from the aqueous humor, the potential for other ocular cells (e.g., RPE) and fluids (e.g., vitreous) to harbor virus, given that EVD survivors may also need surgical intervention for vitreoretinal disease conditions in addition to cataract surgery. 

During the most recent EVD outbreak in the DRC, a randomized controlled trial of therapeutics for EVD was conducted [43]. In this trial, 681 patients were enrolled in the DRC and randomized in a 1:1:1:1 ratio to ZMapp, remdesivir, a single monoclonal antibody Mab114 or the triple monoclonal antibody REGN-EB3 with a primary endpoint of death at 28 days. MAb114 and REGN-EB3 were superior to ZMapp in the reduction of mortality from EVD. Whether these promising results translate to implications for the eye and the reduction of uveitis prevalence remains unknown; however, an improved understanding of the blood–brain barrier and blood–ocular barrier entry of these, and other medications could provide further insight into therapeutics for uveitis associated with EVD [43]. 

## 7. Conclusions and Future Directions

While the West African EVD outbreak and recent DRC outbreaks have provided significant improvements related to our understanding of the prevalence of uveitis and sequelae, as well as their key implications for the vision health of survivors, the mechanisms of ocular disease are the subject of ongoing investigation. The EVICT Study showed that EBOV RNA was not detected in ocular fluid of the majority of patients at 19 months and 34 months post-acute EVD. However, the dynamics of viral entry, persistence and stimulus for the development of lytic infection remains unknown. 

Understanding these dynamics in the eye could provide additional insight into mechanisms of viral escape in other immune-privileged organs, as well as targets for future therapies. The immunologic mechanisms underlying inflammatory eye disease chronicity also warrant exploration, as it remains unclear whether increased prevalence of uveitis over time in EVD survivors is associated with viral persistence or other autoimmune, inflammatory mechanisms. 

Lastly, the recent advances in therapeutics for EVD from the DRC are encouraging in their reduction of mortality. However, the effect of these therapies to other sequelae of EVD including ocular disease and the potential impact on viral persistence in immune-privileged tissue sites warrant further understanding. Investigation in these questions will continue to improve our care of patients with EVD both acutely and during long-term survivorship. 

## Figures and Tables

**Table 1:** Prospective and retrospective studies on eye disease in Ebola virus disease (EVD) survivors.

Author	Location of Study	Type of Study	Number of Cases	Major Clinical Findings
PREVAIL III Study Group [8]	Liberia	Longitudinal	966	26.4% of cases had evidence of uveitis in at least one eye compared to 12% in controls. Prevalence of uveitis increased from baseline to follow-up at 12 months.
Shantha et al. [6]	Liberia	Cross-sectional	96	21 of the 96 cases developed uveitis associated with EVD and 3 developed optic neuropathy associated with EVD. Of the eyes with uveitis, 38.5% had a visual acuity (VA) > 20/400. Anterior, posterior uveitis, and panuveitis were observed
Mattia et al. [15]	Sierra Leone	Cross-sectional	277	Of the 277 cases, ocular complications observed were uveitis (18%), blurry vision (38%), light sensitivity (31%), and foreign body sensation (25%), among others.
Tiffany et al. [5]	Sierra Leone	Prospective	166	166 cases enrolled with 56.6% having ocular complications, the most common being uveitis (34%) in EVD survivors aged 16-30. Anterior uveitis (62%), bilateral uveitis (59%), and panuveitis (21%) were most often encountered. Unilateral worsening of visual acuity in 4 patients, but 9 had improved visual acuity.
Steptoe et al. [16]	Sierra Leone	Case-control	82	82 cases (EVD survivors) enrolled with 75.6% having a Snellen VA of ≤20/25. 7.4% of cases had unilateral white cataracts, and 14.6% had a new retinal lesion.
Shantha et al. [17]	Sierra Leone	Cross-sectional	50	50 cases enrolled (median Snellen VA 20/320—hand motions), 1 with eye pain due to uveitis, 1 with a subluxated lens, 2 with uveitis, and 46 with visually significant cataract. All 50 had a negative RT-PCR test for EBOV RNA within their intraocular fluid
Varkey et al. [9]	Sierra Leone	Case report	1	An EVD survivor demonstrated acute hypertensive anterior uveitis, progressing to severe sight-threatening panuvietis associated with Ebola virus persistence in the aqueous humor.
